# On the interplay between hypothermia and reproduction in a high arctic ungulate

**DOI:** 10.1038/s41598-020-58298-8

**Published:** 2020-01-30

**Authors:** Niels M. Schmidt, Carsten Grøndahl, Alina L. Evans, Jean-Pierre Desforges, John Blake, Lars H. Hansen, Larissa T. Beumer, Jesper B. Mosbacher, Mikkel Stelvig, Eva M. Greunz, Marianna Chimienti, Floris M. van Beest

**Affiliations:** 1 Department of Bioscience, Aarhus University, Roskilde, Denmark; 2 Arctic Research Centre, Aarhus University, Aarhus, Denmark; 3 Centre for Zoo and Wild Animal Health, Copenhagen Zoo, Frederiksberg, Denmark; 4 Department of Forestry and Wildlife Management, Inland Norway University of Applied Sciences, Elverum, Norway; 5 Animal Resources Center, University of Alaska Fairbanks, Fairbanks, USA; 6 Department of Ecosystem and Public Health, University of Calgary, Calgary, Canada

**Keywords:** Ecology, Physiology

## Abstract

For free-ranging animals living in seasonal environments, hypometabolism (lowered metabolic rate) and hypothermia (lowered body temperature) can be effective physiological strategies to conserve energy when forage resources are low. To what extent such strategies are adopted by large mammals living under extreme conditions, as those encountered in the high Arctic, is largely unknown, especially for species where the gestation period overlaps with the period of lowest resource availability (i.e. winter). Here we investigated for the first time the level to which high arctic muskoxen (*Ovibos moschatus*) adopt hypothermia and tested the hypothesis that individual plasticity in the use of hypothermia depends on reproductive status. We measured core body temperature over most of the gestation period in both free-ranging muskox females in Greenland and captive female muskoxen in Alaska. We found divergent overwintering strategies according to reproductive status, where pregnant females maintained stable body temperatures during winter, while non-pregnant females exhibited a temporary decrease in their winter body temperature. These results show that muskox females use hypothermia during periods of resource scarcity, but also that the use of this strategy may be limited to non-reproducing females. Our findings suggest a trade-off between metabolically-driven energy conservation during winter and sustaining foetal growth, which may also apply to other large herbivores living in highly seasonal environments elsewhere.

## Introduction

Shortage of forage resources is a major energetic bottleneck for animals living in highly seasonal environments. While some species migrate to avoid such conditions, sedentary species rely on behavioural and physiological adaptations to conserve energy during the lean period. In mammals, behavioural adaptations may involve seasonal changes in locomotor activity and basking^1^. Physiological adaptations include lowered metabolic rate and lowered body temperatures to conserve energy in response to adverse environmental conditions, ranging from daily torpor to true hibernation^2^, and a variety of such energy conserving strategies has been observed across a variety of species, including ungulates^1,3–5^. Such energy-conserving strategies may, however, depend on intrinsic condition and/or reproductive status of the individual. Indeed, the need for foetal homeothermy in most mammals^6^ may be a decisive factor whether to enter hypothermia or not. In smaller mammals, such as bats, simultaneous gestation and lowered body temperatures is common^7^, but has also been reported for a variety of other species, including sheep^6^, goats^8^ and lemurs^9^. However, it remains unclear the extent to which this strategy can also be adopted by large (>200 kg) animals living in extremely cold and harsh environments.

Few studies have examined the interplay between reproduction and body temperature variability in large, wild mammals. For example, pregnant African lions (*Panthera leo*) lower and stabilize their body temperature (hereafter T_b_) to avoid episodes of hyperthermy^10^, and wolverines (*Gulo gulo*)^11^ also lower their T_b_ during pregnancy despite living in cold environments, whereas hibernating bears exhibit a high degree of homeothermy during pregnancy^12,13^. Also Svalbard reindeer (*Rangifer tarandus platyrhynchus*) lower their T_b_ in winter, and some differences in T_b_ patterns between reproductive and non-reproductive reindeer has been reported^5^, but the linkage between T_b_ and reproductive status remains unclear.

**Figure 1:**
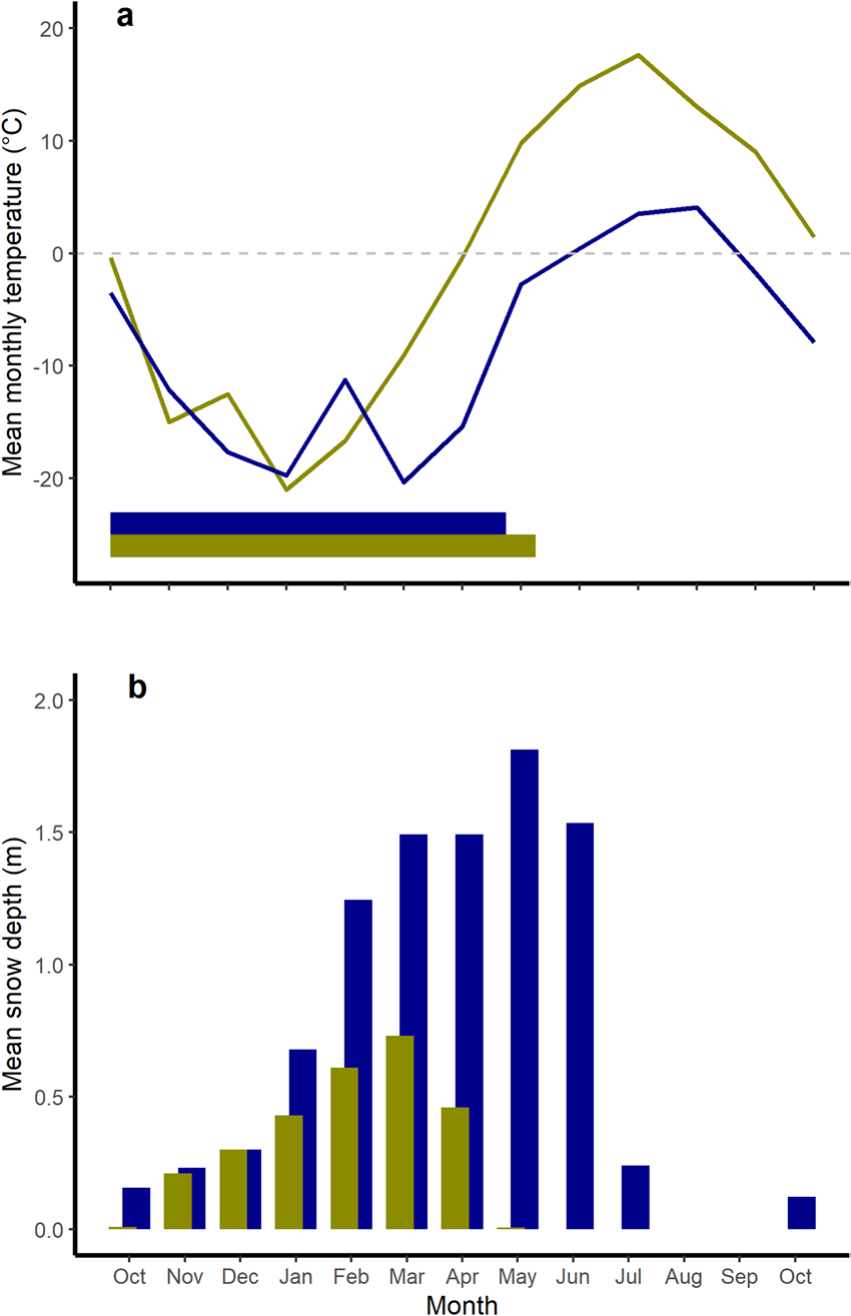
Ambient climatic conditions during the study period (October 2017 to October 2018) at Zackenberg, Greenland (wild population; blue) and Fairbanks, Alaska (captive population; yellow), with (**a**) showing the mean monthly air temperature and (**b**) showing the mean monthly snow depths at two sites. Horizontal bars in (**a**) indicate the approximate gestation period for muskoxen at the two sites. Temperature for Zackenberg were available from Greenland Ecosystem Monitoring (http://data.g-e-m.dk) and for Fairbanks from https://www.usclimatedata.com. Snow depth data were obtained from GeoBasis Zackenberg and for Fairbanks from https://w2.weather.gov/climate/index.php?wfo = pafg.

To gain more insight into the generality of hypothermia as an over-wintering strategy in large mammals and the possible linkages to reproductive status, we target the muskox (*Ovibos moschatus*), the largest herbivore in the Arctic. For muskoxen, the short arctic summer of about three months provides high quality and abundant forage^14^, whereas snow impedes access to the limited plant material available most of the year^15^, making snow conditions a major determinant of muskox habitat use^16^ and population dynamics^17^. During winter, muskoxen rely heavily on energy reserves accumulated over the previous snow-free period^18^. To conserve energy in this extreme environment, we expect muskoxen to enter hypothermia, at least during parts of the winter. Indeed, previous studies have documented lower organ weights and energy expenditure in muskoxen in winter, suggesting lower maintenance costs and down-regulated metabolism during the lean period^18,19^. However, we also hypothesize that hypothermia as an over-wintering strategy in free-ranging muskoxen may interfere with maintenance of foetal growth. As a capital breeder^20^, muskoxen must rely on their energy reserves to cover maintenance and costly reproductive needs as the food-constrained period coincides with the c. 235 day gestation period^21^, but also early lactation (see Table 1 and Fig. 1), and thus likely to have an energy conserving strategy in winter. To examine the interplay between hypothermia and reproduction, we measured core T_b_

**Table 1:** Metadata for all muskox at the time of handling, including their pregnancy status and fate during the study period.

Group	Collar ID	Animal ID	At capture			In study period	
			Body mass (kg)	Pregnancy^b^	Calf at heel?	Date of parturition^c^	Date of death^d^
Survived^a^, birth (wild)	26146	42	201	Presumably negative	No	2018-04-16	Survived
	26147	34	204	Positive	No	2018-03-25	Survived
	26148	43	214	Positive	No	2018-04-25	Survived
Survived, birth (captive)	26150	Kumquat	235	Positive	No	2018-05-07	Survived
	26151	Jenny	242	Positive	No	2018-04-29	Survived
	26152	Yakutia	244	Positive	No	2018-04-28	Survived
Survived^a^, no birth (wild)	26143	30	224	Presumably negative	Yes	No birth event	Survived
	26144	41	236	Presumably negative	Yes	No birth event	Survived
Died^a^, no birth (wild)	26145	23	217	Positive	No	No birth event	2018-05-23^e^
	26149	44	202	Presumably negative	No	No birth event	2018-06-02^e^

Notes: ^a^Based on GPS location data and ground observations, ^b^Based on ultrasound examinations and analyses of serum samples, ^c^Based on VIT readings, ^d^Based on VIT readings and GPS location data, ^e^The carcass of muskox #26145 was located and examined two days after death. At that time, the weight of the still intact carcass was 97 kg, corresponding to a 55% loss of body mass since autumn. No evidence of the foetus was found. Muskox #26149 was confirmed dead, but no body mass could be obtained.

in muskoxen with different reproductive status. Faced with the severe resource limitations during winter and the concurrent need for pregnant females to support foetal growth, we expect muskoxen to conserve energy through lowered body temperatures, but also expect that the level of hypothermia to depend on reproductive status, such that non-pregnant females reduce body temperatures more than pregnant females.

## Results

**Figure 2:**
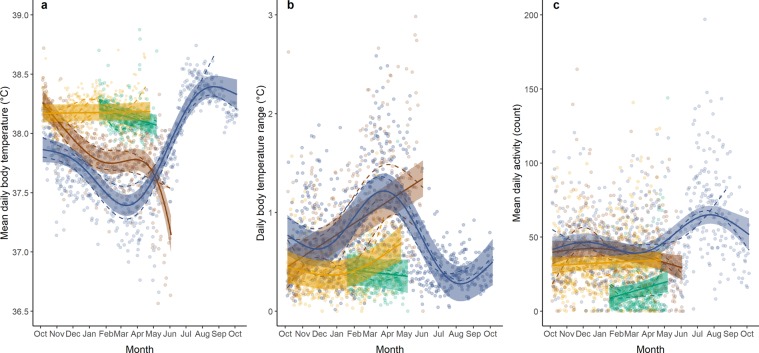
Body temperature and activity profiles of female muskoxen from October 2017 to October 2018. Colours indicate female muskoxen that survived and gave birth in the wild (orange; n = 3) or in captivity (green; n = 3), survived but did not reproduce (blue; n = 2), or died during winter (brown; n = 2): (**a**) mean daily core body temperature (°C), (**b**) daily body temperature range (°C), and (**c**) mean daily activity (count). Dots indicate the individual daily values, dashed lines are the smoothed curves for each muskox individual, while full lines show the predicted curves for each group and the corresponding 95% confidence intervals from the Generalized additive mixed models.

Muskox core T_b_ varied markedly between muskox reproductive groups and across the study period (Fig. 2a). Mean daily T_b_ of pregnant muskoxen that ultimately gave birth were stable during the gestation period, particularly for the wild females (Fig. 2a). In contrast, wild muskoxen that lived through the winter and did not reproduce exhibited a steady decline in core T_b_ until late winter, at which point mean daily T_b_ was about 0.8 °C lower than in pregnant females. After that, T_b_ increased to peak around mid-summer at temperature levels above those observed for all muskox groups in autumn the year before (2017) (Fig. 2a). After the peak, T_b_ of non-reproducing females declined towards the level of pregnant females from autumn 2017 (Fig. 2a). T_b_ of muskoxen that eventually died exhibited a gradual decline throughout winter until death (Fig. 1a). While we do not know the cause of death of these two animals, we do know that at the time of death, one animal had lost more than half of its autumn body mass (Table 1), testifying to a severe depletion of body reserves.

At the time of collaring, T_b_ of muskoxen that later gave birth and those who died without giving birth (Table 1) were similar (Fig. 2a), while non-pregnant muskoxen with a calf at heel (Table 1) had slightly lower mean daily T_b_ (approximately 0.3 °C; Fig. 2a). Given that we evaluated muskox pregnancy status very early in the gestation period^22^, and assuming alternate year breeding^23^, it seems likely that both animals that died in winter (Table 1) were pregnant at the time of collaring.

Pregnant muskoxen exhibited the smallest daily T_b_ ranges (both wild and captive) compared to the other muskox groups (Fig. 2b). Daily T_b_ ranges of captive muskoxen decreased during the gestation period, while all wild muskoxen showed increasing daily T_b_ ranges towards the end of winter (Fig. 2b). For muskoxen that survived throughout the study period, temperature ranges declined again in summer, thus exhibiting a pattern opposite to that of the mean daily T_b_ (Fig. 2a,b). Daily minimum T_b_ in particular, but also daily maximum T_b_, roughly followed the same pattern as the mean daily T_b_ (Supplementary Fig. S1), providing further evidence that pregnant females exhibited a much more stable T_b_ compared to the other groups. All four-hourly body temperature recordings are presented in Supplementary Fig. S2.

Muskox activity patterns were highly variable, both within groups and over the study period (Fig. 2c). Amongst the groups consisting of wild muskoxen, muskoxen that gave birth tended to be the least active (Fig. 2c). Compared to the winter period, the summer period was characterized by high activity levels, at least for non-reproducing females from which data were obtained (Fig. 2c).

Births indicated by expelled VITs were confirmed in all captive muskoxen, and we therefore expect that three birth events happened in the wild as well. Of the three pregnancies in wild muskoxen, only two were detected at the time of collaring (Table 1), and one (#26146) thus represents a false negative pregnancy, either because it was too early to diagnose the gestation or because the female conceived after collaring (Table 1).

## Discussion

Our study clearly shows that T_b_ of female muskoxen in the high Arctic varies markedly across the year, and some females displayed lowered body temperature during periods of resource scarcity. This suggests that muskoxen can adopt hypothermia as an over-wintering strategy to conserve energy. Body temperature and metabolic rate generally correlates well across vertebrate species^24^ and within individuals^25,26^ (but not always^27^), and hypothermia may thus be indicative of hypometabolism. Both hypothermia and hypometabolism have previously been reported for a variety of mammal species^1,3^, including high latitude ungulates^5^, as adaptations to the annual cycle in seasonal environments. However, and more importantly, we revealed a link between T_b_, activity level, and pregnancy status in muskoxen. Without further study we cannot untangle whether these differences in T_b_, and thus likely differences in metabolic rates, are caused by the reproductive status of the female or vice versa, highlighting an important gap in current knowledge that needs further study. Regardless, the differences in T_b_ between pregnant and non-pregnant muskoxen likely result in substantially different metabolic costs^28^.

As hypothesized, pregnant females that carried their foetus to term exhibited a high degree of homeothermy during the gestation period. This pattern resembles that observed in other mammals, such as bears^13^. However, while Thiel *et al*.^11^ also reported more stable T_b_ in pregnant wolverines as compared to non-pregnant ones, they found that wolverines lowered their T_b_ during gestation. These contrasting reports suggest that the use of hypothermia as an over-winter strategy during pregnancy likely depends on species-specific life-history traits, for instance body mass and whether a given species relies on accumulated reserves or continues feeding during gestation. Indeed, in addition to environmental conditions, the degree of heterothermy in mammal species is linked to their evolutionary history^29^.

We observed remarkably similar mean daily T_b_ (and its range) for both captive and wild pregnant muskoxen, despite clear differences in food availability, ambient temperature and snow conditions (Fig. 1). This finding suggests that both groups of pregnant females had sufficient energy reserves to sustain somatic maintenance and foetal growth without lowering their core T_b_, whilst also providing thermal protection for the developing foetus^30^ during the cold arctic winter. Though these reproductively successful wild females were generally the least active in winter compared to the other wild muskoxen, differences were not pronounced and therefore only marginal differences in energy expenditure are to be expected. Nonetheless, the lower activity levels may be seen as a general energy conserving strategy but also indicative of lower need for foraging and relocation activities in winter, further supporting that these individuals were in better condition in winter as compared to the other wild muskoxen, enabling them to both survive and cover gestational costs.

Activity levels and T_b_ of wild non-pregnant muskoxen increased steadily after the winter nadir until mid-summer, where T_b_ reached levels above those observed the previous autumn for any of the muskox groups. This pattern likely reflects increased foraging activities in the summer season to replenish energy reserves^31^. The surviving non-pregnant females all had a calf at heel in the autumn and had thus been nursing the young in the summer before collaring. These females had slightly lower T_b_ compared to both wild and captive reproducing females already in late autumn, and thus already exhibited signs of hypothermia. Whilst the reduction in T_b_ was quite small, this may indicate that these females had insufficient body reserves in autumn to support oestrus, likely due to the high energetic costs of lactation for the calf at heel^32^. In muskoxen, lactation does not preclude pregnancy, but appears limited to those in better body condition^18^. Interestingly, one year after collaring, T_b_ levels of females that were non-pregnant in autumn 2017 reached T_b_ levels of pregnant females. These patterns support previous findings that the high costs associated with pregnancy and lactation^32^ may lead to alternate year breeding in wild muskoxen in northeast Greenland^23^. However, at the time of collaring, the non-pregnant muskoxen were the heaviest among the wild muskoxen (Table 1), suggesting that body mass alone is not the sole determinant of oestrus and that a physiological postpartum (lactational) anoestrus^33^ may contribute to the observed pregnancy patterns.

For both groups of non-reproducing muskoxen in the wild, the rapid T_b_ decline may indicate a starvation-induced temperature drop, as similar T_b_ signatures have been found in other starving animals^28^, including ungulates^34^. However, in contrast to the surviving non-reproducing females, females that eventually died did not manage to reverse the negative temperature development in late winter, and the late drop in T_b_ can be seen as a stress response to severe energy depletion. Though it was evident in all wild muskox groups that daily T_b_ ranges increased during the course of winter, likely induced by energy deficiency^35^, the muskoxen that eventually died exhibited the most dramatic loss of homeothermy with the largest and rapidly increasing daily T_b_ ranges during winter.

In this study we have shown that T_b_ of some muskox females decreased during winter, indicating the use of hypothermia, and potentially also hypometabolism, during the resource-poor and harsh high arctic winter. However, this physiological energy conservation strategy appears restricted to females that do not (successfully) reproduce. Hypothermia may limit overwinter depletion of energy stores and subsequently allow these individuals to reproduce the following year. However, in endothermic animals where T_b_ is generally regulated within a narrow range, heterothermy may come at a cost. In rabbits (*Oryctolagus cuniculus*), increased daily heterothermy resulted in reduced future fitness in terms of fewer litters born^35^. Whether such fitness cost is applicable to other species, such as the muskox, is not known. Interestingly, muskoxen living in more productive areas than high arctic Greenland, may have successive year breeding^23^, indicating that metabolic strategies may be flexible in muskoxen, with hypothermia limited to individuals living in areas with severe energetic constraints in winter, and where current survival may outweigh future fitness costs.

Despite our limited sample size, we posit that the link between reproduction and hypothermia as an overwintering strategy may be due to a trade-off between the need to reduce metabolic costs and the need to maintain foetal growth. We advocate that an intimate link between energy reserves and reproductive status may exists, at least in muskox females^32^ under severe energetic constraints, which can have subsequent consequences on individual energy balance and ultimately population dynamics. While our study has provided the first indications of such a relationship in a wild, large herbivorous mammal, we emphasise that more studies on the linkage between animal T_b_, metabolism and pregnancy, particularly from highly seasonal environments, are needed to further support our claims. Understanding individual energy balances in species occupying highly seasonal environments is a prerequisite for assessing how populations may cope with current and future changes in climatic conditions.

## Methods

### Animal handling

In autumn 2017, we captured seven female muskoxen (Table 1) at Zackenberg in high arctic Greenland (74°28’ N, 20°34’ W) to study their movement patterns^31^. The sedation and handling of muskoxen in this study followed the guidelines of the American Society of Mammalogists^36^. Specific immobilization and handling procedures are described in Grøndahl *et al*.^37^. Approval of experimental protocols and ethical aspects were granted under the research permits issued by the Greenland Government, Ministry of Domestic Affairs, Nature and Environment (j.no. G17-14) and by the Institutional Animal Care and Use Committee at University of Alaska (#1165790). All females handled were adults (4 years or more; aged based on horn morphology^38^). Early pregnancy status was determined using intravaginal ultrasound and ELISA on serum samples^22^. To obtain information about time and place of parturition and physiological parameters^39,40^, we deployed sterilized vaginal implant transmitters (VIT; model 8019, Vectronic Aerospace GmbH, Germany). VITs consisted of a tubus (measuring 80 × 19mm), an antenna (195 mm) and wings (spanning 144 mm) and weighed approximately 48 g. VITs were programmed to log T_b_ (accuracy 0.1 °C) and accumulated activity every 4 hours (ranging between 1 and 255 (minimum and maximum activity, respectively)). Before deployment, VIT calibration in ice water showed no need for VIT-specific adjustments. As VITs were not recovered after deployment, we were unable to check for drift in temperature measurements. Temperature recordings were transmitted to a GPS collar (VERTEX PLUS-6 Collar, Vectronic Aerospace GmbH, Germany; weight app. 1.7 kg, corresponding to less than 1% of average muskox body mass), via an external FIWI repeater built into the battery housing of the collar. From the collar, VIT data were transmitted daily to a remote server via an Iridium satellite unit. In the event of parturition, the loss of the VIT would immediately trigger an alert message via the collar. For calibration and comparison, we deployed the same equipment on three pregnant muskoxen kept in captivity at the Large Animal Research Station at the University of Alaska Fairbanks in January 2018. The three muskoxen were fed ad libitum during gestation and expelled the VITs during calving in April-May 2018 (Table 1).

### Statistical analysis

We first assigned all muskoxen to groups according to their reproductive state and survival during the study period: (1) wild pregnant females that calved and lived through the study period (n = 3), (2) wild non-pregnant females that lived through the study period (n = 2), (3) captive pregnant females that calved and lived through the study period (n = 3), and (4) wild females that died during the study period without calving (n = 2) (Table 1). We excluded all T_b_ recordings from the day of deployment, from expelled VITs and after a female had died. For the captive muskoxen, we also excluded one day of observations (4 April 2018) when these animals were vaccinated. For each individual, we then calculated i) mean daily T_b_, ii) daily T_b_ range (T_b*max*_ - T_b*min*_)_,_ and iii) mean daily activity. We analysed these response variables individually using Generalized additive mixed models (GAMM), using the function “*gamm*” from the R package “*mgcv*”^41^. We modelled each response variable as a function of group and day of year (DOY; with smooth term added), with individual ID as random factor and with a first-order autoregressive function within individuals to account for temporal autocorrelation. Model fitted residuals were inspected visually and no transformation of data was needed. Statistical differences in the trajectory of daily core T_b_ and activity measures is indicated by non-overlapping 95% confidence intervals in Fig. 2 and Supplementary Fig. S1.

## Supplementary information

Supplementary figures.

## Data Availability

Supporting data can be found in 10.5281/zenodo.3584998.

## References

[1] Signer C, Ruf T, Arnold W (2011). Hypometabolism and basking: the strategies of Alpine ibex to endure harsh over-wintering conditions. Funct. Ecol..

[2] Ruf T, Geiser F (2015). Daily torpor and hibernation in birds and mammals. Biol. Rev..

[3] Riek A (2017). Seasonal changes in energy expenditure, body temperature and activity patterns in llamas (*Lama glama*). Sci. Rep. -UK.

[4] Arnold W (2004). Nocturnal hypometabolism as an overwintering strategy of red deer (*Cervus elaphus*). Am. J. Physiol. -Reg. I..

[5] Arnold W (2018). Circadian rhythmicity persists through the Polar night and midnight sun in Svalbard reindeer. Sci. Rep. -UK.

[6] Laburn HP, Faurie A, Goelst K, Mitchell D (2002). Effects on fetal and maternal body temperatures of exposure of pregnant ewes to heat, cold, and exercise. J. Appl. Physiol..

[7] McAllan BM, Geiser F (2014). Torpor during Reproduction in Mammals and Birds: Dealing with an Energetic Conundrum. Integr. Comp. Biol..

[8] Faurie AS, Mitchell D, Laburn HP (2001). Feto-maternal relationships in goats during heat and cold exposure. Experimental Physiology.

[9] Canale CI, Perret M, Henry PY (2012). Torpor use during gestation and lactation in a primate. Naturwissenschaften.

[10] Trethowan Paul D., Hart Tom, Loveridge Andrew J., Haw Anna, Fuller Andrea, Macdonald David W. (2016). Improved homeothermy and hypothermia in African lions during gestation. Biology Letters.

[11] Thiel A (2019). Effects of reproduction and environmental factors on body temperature and activity patterns of wolverines. Front Zool.

[12] Friebe A (2014). Factors affecting date of implantation, parturition, and den entry estimated from activity and body temperature in free-ranging brown bears. Plos One.

[13] Shimozuru M (2013). Pregnancy during hibernation in Japanese black bears: effects on body temperature and blood biochemical profiles. J. Mammal..

[14] Mosbacher JB, Kristensen DK, Michelsen A, Stelvig M, Schmidt NM (2016). Quantifying muskox plant biomass removal and spatial relocation of nitrogen in a High Arctic tundra ecosystem. Arct. Antarct. Alp. Res..

[15] Schmidt NM, Mosbacher JB, Vesterinen EJ, Roslin T, Michelsen A (2018). Limited dietary overlap amongst resident Arctic herbivores in winter: complementary insights from complementary methods. Oecologia.

[16] Beumer LT, van Beest FM, Stelvig M, Schmidt NM (2019). Spatiotemporal dynamics in habitat suitability of a large Arctic herbivore: environmental heterogeneity is key to sedentary lifestyle. Global Ecology and Conservation.

[17] Schmidt NM, Pedersen SH, Mosbacher JB, Hansen LH (2015). Long-term patterns of muskox (*Ovibos moschatus*) demographics in high arctic Greenland. Polar Biol..

[18] Adamczewski JZ, Flood PF, Gunn A (1997). Seasonal patterns in body composition and reproduction of female muskoxen (*Ovibos moschatus*). J. Zool..

[19] Lawler JP, White RG (1997). Seasonal changes in metabolic rates in muskoxen following twenty-four hours of starvation. Rangifer.

[20] Kerby J, Post E (2013). Capital and income breeding traits differentiate trophic match-mismatch dynamics in large herbivores. Phil. Trans. R. Soc. B.

[21] Pharr JW, Rowell JE, Flood PF (1994). Fetal growth in muskoxen determined by transabdominal ultrasonography. Can. J. Vet. Res..

[22] Greunz EM (2018). Evaluation of two Enzyme-linked Immuno-sorbent Assays measuring Pregnancy-associated Glycoproteins in the Blood of Muskoxen (*Ovibos moschatus)*. J. Zoo Wildlife Med..

[23] Thing H, Klein DR, Jingfors K, Holt S (1987). Ecology of Muskoxen in Jameson Land, Northeast Greenland. Holarctic Ecol..

[24] Clarke A, Rothery P, Isaac NJB (2010). Scaling of basal metabolic rate with body mass and temperature in mammals. J. Anim. Ecol..

[25] Schmidt-Nielsen K, Crawford EC, Newsome AE, Rawson KS, Hammel HT (1967). Metabolic rate of camels: effect of body temperature and dehydration. Am. J. Physiol..

[26] Heldmaier G, Ruf T (1992). Body temperature and metabolic rate during natural hypothermia in endotherms. J. Comp. Physiol. B.

[27] Tøien Ø (2011). Hibernation in black bears: independence of metabolic suppression from body temperature. Science.

[28] McCue MD (2010). Starvation physiology: Reviewing the different strategies animals use to survive a common challenge. Comparative Biochemistry and Physiology Part A: Molecular & Integrative Physiology.

[29] Boyles JG (2013). A global heterothermic continuum in mammals. Global Ecology and Biogeography.

[30] Laburn, H., Faurie,A., Mitchell,D., & Cronje,P.B. In *Ruminant physiology: digestion, metabolism, growth and reproduction* (ed. Cronje,P.B.) 295–310 CABI Publishing, New York, 2000).

[31] Schmidt NM (2016). Ungulate movement in an extreme seasonal environment: Year-round movement patterns of high-arctic muskoxen. Wildlife Biol..

[32] Desforges J-PW (2019). Quantification of the full lifecycle bioenergetics of a large mammal in the high Arctic. Ecol. Model..

[33] Stevenson, J. S. In Encyclopedia of reproduction (eds. Knobil,E. & Neill, J. D.) 954–963 (Raven Press, New York, 1999).

[34] Turbill C, Ruf T, Mang T, Arnold W (2011). Regulation of heart rate and rumen temperature in red deer: effects of season and food intake. J. Exp. Biol..

[35] Maloney, S. K., Marsh, M. K., McLeod, S. R. & Fuller, A. Heterothermy is associated with reduced fitness in wild rabbits. *Biol. Lett*. **13**, 20170521 (2017). 10.1098/rsbl.2017.0521 PMC5746534 29212751

[36] Sikes RS, Gannon WL (2011). Guidelines of the American Society of Mammalogists for the use of wild mammals in research. J. Mammal..

[37] Grøndahl C (2018). Immobilizing Muskox (*Ovibos moschatus*) under High Arctic Conditions. J. Zoo Wildlife Med..

[38] Olesen CR, Thing H (1989). Guide to field classification by sex and age of the muskox. Can. J. Zool..

[39] Thompson DP (2018). Vaginal implant transmitters for continuous body temperature measurement in moose. Wildl. Soc. Bull..

[40] Johnson BK, McCoy T, Kochanny CO, Cook RC (2006). Evaluation of vaginal implant transmitters in elk (*Cervus elaphus nelsoni*). J. Zoo Wildlife Med..

[41] Wood, S. N. *Generalized additive models: an introduction with R* (Chapman and Hall/CRC, 2017).

