# Supplementary material for: On the interplay between hypothermia and reproduction in a high arctic ungulate
Source: Sci Rep. 2020 Jan 30;10:1514. doi: 10.1038/s41598-020-58298-8 (PMC6992616; doi:10.1038/s41598-020-58298-8)
Supplement: Supplementary file 1 — Supplementary figures. [file 41598_2020_58298_MOESM1_ESM.pdf]

Supplementary material from "On the interplay between hypothermia and reproduction in a high arctic ungulate"

Niels M. Schmidt, Carsten Grøndahl, Alina L. Evans, Jean-Pierre Desforges, John Blake, Lars H. Hansen, Larissa T. Beumer, Jesper B. Mosbacher, Mikkel Stelvig, Eva M. Greunz, Marianna Chimienti and Floris M. van Beest

Daily maximum and minimum body temperatures

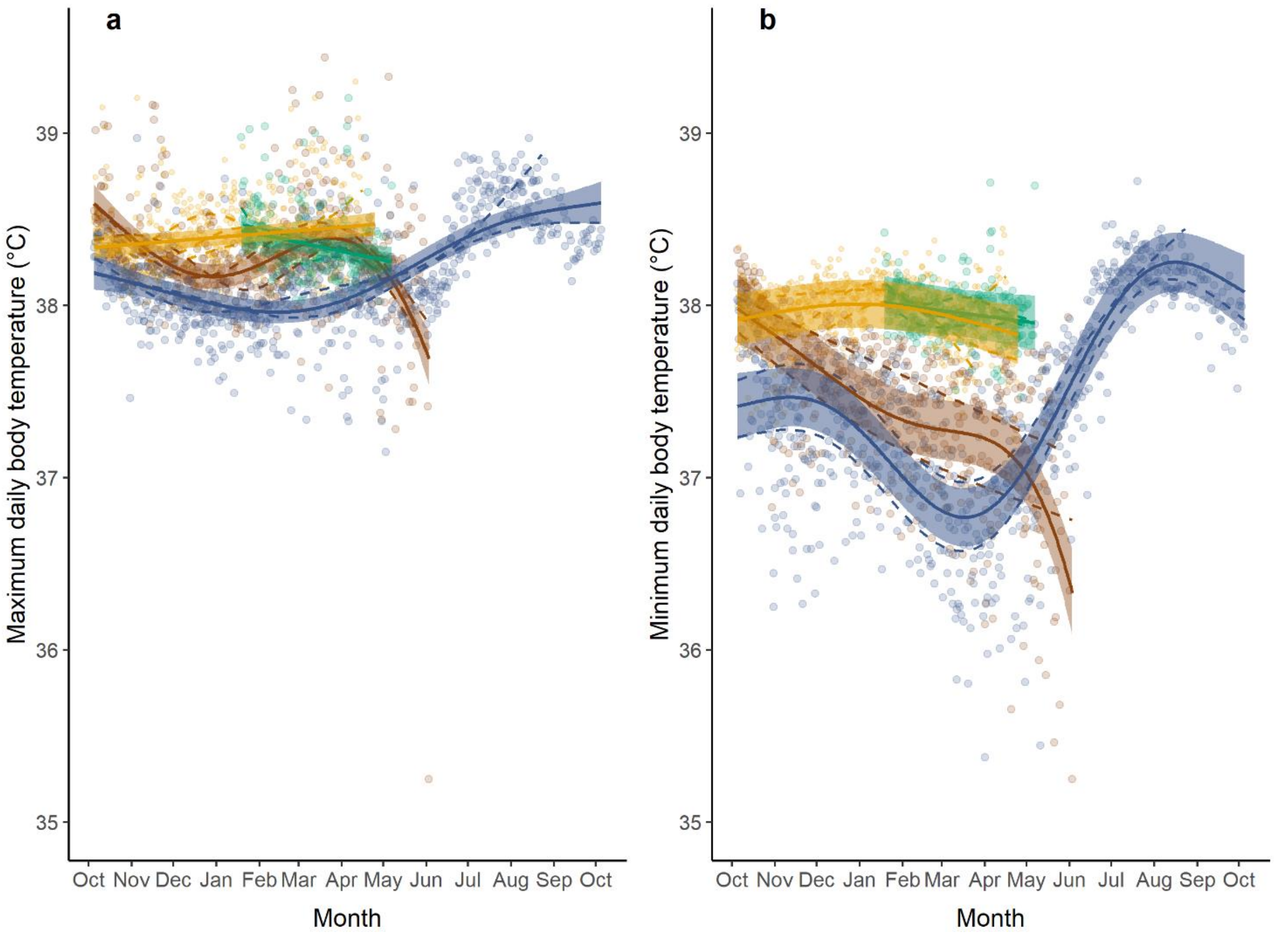

Figure S1. Muskox body temperatures during the study period (October 2017 to October 2018), with **a**) showing maximum daily body temperatures and **b**) showing minimum daily body temperatures. Dots indicate the individual daily values, stippled lines are the smoothed curves for each muskox individual, while full lines show the predicted curves for each group and the corresponding 95% confidence intervals from the Generalized additive mixed models.

Raw body temperature recordings for the four groups

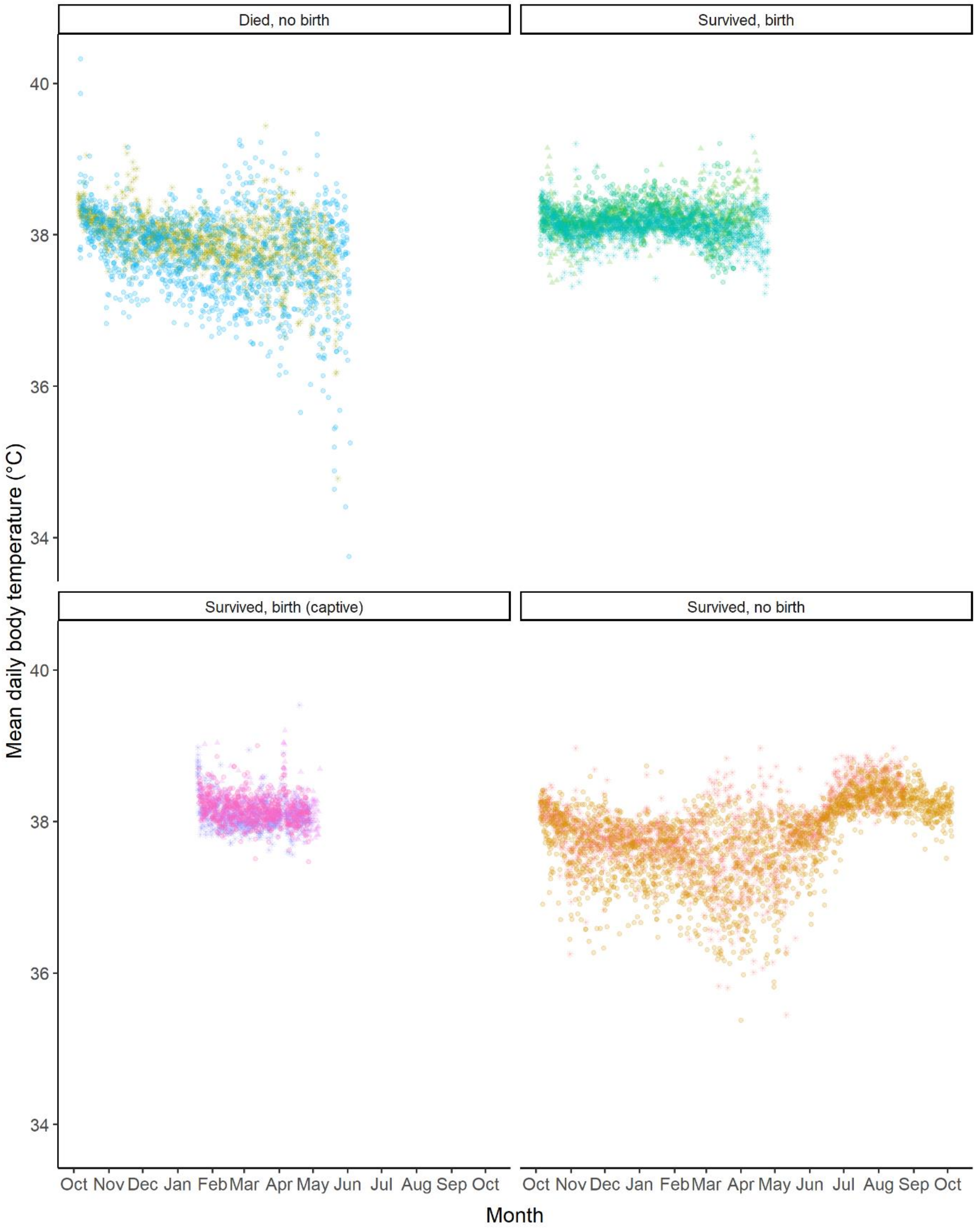

Figure S2. Four-hourly body temperature (°C) recordings from seven wild female muskoxen in NE Greenland and three captive female muskoxen in Alaska from October 2017 to October 2018, broken down into the four muskox groups: Female muskoxen that died during winter (n=2), survived and gave birth in the wild (n=3) or in captivity (n=3), or survived but did not reproduce (n=2).
